# Utilization of the clinical laboratory for the implementation of concussion biomarkers in collegiate football and the necessity of personalized and predictive athlete specific reference intervals

**DOI:** 10.1186/s13167-016-0050-x

**Published:** 2016-01-27

**Authors:** Stefanie Schulte, Natalie N. Rasmussen, Joseph W. McBeth, Patrick Q. Richards, Eric Yochem, David J. Petron, Frederick G. Strathmann

**Affiliations:** 1 Department of Psychology, University of Utah School of Medicine, Salt Lake City, UT 84108 USA; 2 Department of Exercise and Sport Science, University of Utah School of Medicine, Salt Lake City, UT 84108 USA; 3 Department of Pathology, University of Utah School of Medicine, 500 Chipeta Way mail code 115, Salt Lake City, UT 84108 USA; 4 Department of Athletics, University of Utah School of Medicine, Salt Lake City, UT 84108 USA; 5 Department of Orthopaedics, University of Utah School of Medicine, Salt Lake City, UT 84108 USA; 6 ARUP Institute for Clinical and Experimental Pathology, ARUP Laboratories, Salt Lake City, UT 84108 USA

**Keywords:** Sports-related concussion, Biomarker panel, Predictive diagnostics, Blood test, Collegiate athletes, Football, Traumatic brain injury, S100B, NSE

## Abstract

**Background:**

A continued interest in concussion biomarkers makes the eventual implementation of identified biomarkers into routine concussion assessment an eventual reality. We sought to develop and test an interdisciplinary approach that could be used to integrate blood-based biomarkers into the established concussion management program for a collegiate football team.

**Methods:**

We used a CLIA-certified laboratory for all testing and chose biomarkers where clinically validated testing was available as would be required for results used in clinical decision making. We summarized the existing methods and results for concussion assessment across an entire season to identify and demonstrate the challenges with the eventual integration of a parallel process using blood-based tests for concussion management. We analyzed the results of the biomarkers chosen for trends consistent with the outcome assessments provided from the current concussion management protocols.

**Results:**

Baseline samples were collected with three additional post-concussion samples collected at three separate time points from players with a diagnosed concussion (*n* = 12). A summary of results from currently used concussion assessment tools were compared to the representative biomarkers S100B and NSE results. Nine sport-related concussions occurred during practice and three during play. For S100B, 50 % had follow-up testing results lower than the post-injury result. In contrast, 92 % of NSE follow-up results were lower than post-injury. One hundred percent of the results for S100B and NSE were within the athlete-derived reference intervals upon return-to-play and season end.

**Conclusions:**

The reported workflow provides a framework for the eventual implementation of biomarkers for concussion assessment into existing assessment protocols and strengthens the need for reliance on clinical laboratory testing. Athlete-specific reference intervals will be required to adequately interpret results.

**Electronic supplementary material:**

The online version of this article (doi:10.1186/s13167-016-0050-x) contains supplementary material, which is available to authorized users.

## Background

Sport-related concussion (SRC) continues to receive tremendous global attention. It has been estimated that up to 3.8 million SRCs occur in the USA each year [1]. Approximately 5.8 % of all collegiate athletic injuries are SRCs with higher rates in contact sports such as football [2]. Recent findings suggest that collegiate football players who sustained one SRC in a season are more likely to sustain a second concussion in the same season compared to uninjured players. Additionally, collegiate football players are more likely to return-to play (RTP) early following concussion than athletes in other sports [3]. Accordingly, collegiate football players represent an important at risk population regarding concussion assessment and treatment.

Given the amount of SRC in collegiate football and the potential risk for long-term consequences associated with RTP, greater focus on the management and monitoring of SRC from multiple aspects is required and ongoing. Since various concussion laws endeavor to increase athletes’ protection from the potential consequences of SRC, the Sports Concussion Management Policy of Utah High School Activities Association (UHSAA) serves as a standard for Utah’s concussion management in high schools though collegiate athletics are arguably held to higher standards [4]. This policy recommends computerized neuropsychological assessment (CNA) which includes a pre‐season baseline to allow comparison of concussed athletes’ performance to their baseline values. Accordingly, the Concussion Program of the University of Utah, Department of Athletics implemented CNA as part of a comprehensive physical and neurocognitive assessment for Utah’s collegiate football players [5]. Furthermore, the neurophysiological test Balance Error Scoring System (BESS) and the Sport Concussion Assessment Tool 2 ((SCAT2) at the time of manuscript preparation; currently superseded by SCAT3) are standardized sideline tools and follow-up evaluations for concussed football players. Additionally, a concussion symptom chart/checklist based on the University of Utah Sports Medicine Concussion Management Plan is used to monitor athletes’ medical outcome. Even though these neurophysiological tests are relatively well-established testing methods in concussion management, recent studies have expressed concerns about the reliability and validity of neuropsychological tests for SRC assessment [6, 7]. Controversies regarding appropriate concussion diagnosis and management could potentially lead to premature RTP decisions [8]. Determining the RTP accurately can be challenging due the variability in presentation of concussion symptoms, and a lack of objective data indicating when full recovery has occurred [9, 10]. Accordingly, the accurate diagnosis of SRC and determination of RTP criteria are crucial for a collegiate football player to recover from SRC.

The need for accurate diagnosis and management of SRC in an unbiased manner has resulted in efforts to identify blood-based biomarkers which are capable of documenting quantifiable biological changes directly related to physiological trauma [11, 12]. The clinical use of blood-based biomarkers in addition to existing SRC assessment presents multiple advantages. Firstly, blood-based biomarker measurement is minimally invasive and causes less cost for processing and analysis. Secondly, an accurate determination or exclusion of SRC would assist in the decision of whether further brain imaging or following-up testing is necessary. Thirdly, if appropriate biomarker concentrations could be correlated with the extent of SRC and progression of the injury, results could be used to objectively assess recovery time and RTP [13]. The majority of proposed biomarkers for concussion assessment have been identified and tested in the research setting. However, two extensively studied protein biomarkers for brain injury, S100B and neuron-specific enolase (NSE) [14], are available clinically. S100B concentrations of less than 0.1–0.12 μg/l have been shown to be a reliable tool to predict a normal CT scan and to support the clinician’s decision not to perform CT imaging [15, 16]. Furthermore, serum S100B concentrations greater than 0.027 ng/mL were found to significantly correlate with more than 90% of abnormal cerebrospinal-fluid albumin quotient results, the current gold standard for determining blood-brain barrier dysfunction (BBBD) [17]. Thus, the measurement of S100B is used in emergency room settings to rule out concussions [15, 18]. Serum NSE levels of more than 16.32 μg/L are considered to be pathological [19]. NSE is a marker which directly reflects traumatic damage of the neurons [20]. Also, NSE has been shown to be 87 % sensitive and 82.1 % specific in predicting poor neurologic outcome in concussed patients [21]. In terms of predicting intracranial pathology and long lasting neurocognitive disability, early concentration peaks of both S100B and NSE have been shown to be sensitive indicators following concussion and BBBD [14, 22]. Thus, the markers S100B and NSE have demonstrated reasonable prognostic value for concussion management despite the existence of conflicting data [22, 23].

Given the potential value and promise of biomarker integration into athletic concussion assessment, the development of a successful workflow for their inevitable integration is crucial. The use of blood-based biomarkers in SRC management also implies the practicality of venipuncture and blood processing in situations and under circumstances that are not in line with the WHO guidelines [24]. Errors and delays in the blood collection and processing system can affect the diagnostic process [25]. In the present study, we sought to develop and test an interdisciplinary approach for the integration of blood-based biomarkers into the established concussion management program for the collegiate football team at the University of Utah using S100B and NSE as example biomarkers. The selection of S100B and NSE was based largely on their availability as clinically validated tests that could be conducted in a CLIA-certified central laboratory facility in contrast to a research setting. Herein, we present an established and successful interdisciplinary work flow for blood collection, on-site sample processing, and central laboratory testing for the duration of an entire collegiate football season. The included data provide a summary of the concussion assessment tools available at the time the study was conducted and demonstrate the successful integration of biomarker monitoring into collegiate athletics.

## Methods

This project and its protocols were approved by the University of Utah Institutional Review Board (IRB #00061977). The demographic summary for the included athletes (all male) was as follows: age range, 18 to 26 years; mean age, 21 years. For the baseline venipuncture, athletes were separated into two groups of approximately 60 athletes with the baseline collection occurring within 2 h post-physical activity that did not include full contact play. Five certified phlebotomists were present for the baseline venipuncture with each group of athletes taking approximately 1 h to complete. Individual venipuncture kits were organized to include all necessary materials for the venipuncture, and similar draw kits were used for the remaining venipunctures throughout the season (Additional file 1: Table S1). For post-injury venipunctures, two assistant athletic trainers with access to the athletes conducted phlebotomy training and met the required minimum for the observed venipunctures with certified phlebotomists prior to the start of the season. A Champion E-33 Series centrifuge (Ample Scientific LLC, Norcross, GA) was purchased for sample processing. Sample collection procedures were in line with those established for clinical testing including separation of the serum from the cells within 2 h and stored refrigerated until next day pick-up for transfer to the laboratory.

Samples were collected in red-top tubes, allowed to clot, and were separated from the cells within 2 h of being drawn. Aliquots were stored at −70 °C until testing. CanAg® NSE and S100B enzyme-linked immunosorbent assay kits (Fujirebio Diagnostics, Inc.,Göteborg, Sweden) were used following manufacturer directions provided in the package inserts, and testing was conducted in a CLIA-certified laboratory environment consistent with established laboratory Standard Operating Procedure. For the NSE test, 96-well plates were washed with a buffered wash solution, then 25 μL of calibrators, QC material, and subject samples were added to each well in duplicate as per manufacturer recommendation. A 100 μL of antibody solution was added to the wells and the plate was incubated, shaking for 1 h at room temperature. Following incubation, wells were rinsed with the buffered wash solution and 100 μL of substrate was added. After 30 min of incubation at room temperature, 100 μL of stop solution was added to each well. The plates were read at 405 nm using a Spectramax® Plus384 spectrophotometer (Molecular Devices Corp., Sunnyvale, CA). A calibration curve of absorbance (OD) versus concentration (μg/L) using a quadratic fit was generated by the plate reader software. Concentrations of QC and subject samples were automatically determined from the curve by the software. For the S100B test, 96-well plates were rinsed with a buffered wash solution. Fifty microliter of calibrators, QC material, and subject samples were added to each well in duplicate, followed by 100 μL of biotin anti-S100. The plate was shaken at room temperature for 2 h, and then rinsed with the wash buffer solution. A 100 μL of tracer working solution was added to each well and the plate was incubated for 1 h, shaking at room temperature. After incubating, wells were rinsed with buffered wash solution and 100 μL of substrate was added. After 30 min, 100 μL of stop solution was added and plates were read at 405 nm using the same spectrophotometer listed above. A calibration curve of absorbance (OD) versus concentration (μg/L) using a quadratic fit was generated by the plate reader software. Concentrations of QC and subject samples were automatically determined from the curve by the software. Analytical limits of quantification are 1–150 μg/L for NSE and 12–3500 ng/L for S100B.

Duplicate sample results all had CVs <15 %; no repeats were necessary. Two levels of NSE QC material were pooled in house using de-identified patient samples previously tested for NSE. CanChek Tumor Marker Control Sera Levels 1 and 2 (IBL America, Inc.) were used as QC material for S100B. QC quantitated within two SDs of the mean established by the clinical laboratory for the current lots of material.

### Concussion assessment tools

RTP decision management focuses mostly on signs and symptoms of neuropsychological test batteries [26, 27]. In accordance with Shane et al. [28], we chose to use the symptoms sections of the neuropsychological assessment tools. The symptom charts of SCAT2, CNA, and the University of Utah Sports Medicine Concussion Symptom Checklist compromise 22 similar symptoms with a symptom scoring from 0 (none) to 6 (severe).

### SCAT2

The Sport Concussion Assessment Tool 2 (SCAT2) was developed to replace the original SCAT during the “3rd International Conference on Concussion in Sport” in 2008 and was in place at the time of manuscript preparation [29]. The SCAT2 was widely established as the standardized technique to evaluate, assess, and manage SRC for individuals older than 10 years with the end goal of safely returning the athlete to play [30]. SCAT2 enabled medical and health professionals to assess concussion in a standardized way by evaluating seven areas including symptoms, physical signs, Glasgow Coma Scale, sideline assessment using Maddocks score, cognitive assessment, balance, and coordination. The symptom chart of SCAT2 required the participant to score him/herself on 22 symptoms on a scale from 0 (none) to 6 (severe) based on how he or she is feeling. A total number of symptoms (max. 22) and a symptom severity score (max. 22 * 6 = 132) is calculated. Furthermore, the participant was asked if physical or mental activity worsens the symptoms. In the event that the tester knows the participant well prior to the injury, he or she was asked how different the participant is acting compared to his or her usual self. The score of the symptom chart influences the total score of SCAT2. The SCAT3 is currently in use, and key differences are described elsewhere [30].

### Computerized neuropsychological assessment (CNA)

Immediate Post-Concussion Assessment and Cognitive Testing (ImPACT) is the most widely used computerized neuropsychological test in the USA [31]. The ImPACT test compromises four sections including a demographic profile and health history questionnaire, current concussion symptoms and conditions, baseline and post-injury neurocognitive tests, and a graphic display of ImPACT test scores [32]. As in SCAT2, the symptom chart of ImPACT covers 22 symptoms with most of the symptoms being congruent and ranks the symptoms on a scale from 0 being “symptom free” to 6 being “severe”.

### University of Utah Sports Medicine Concussion Symptom Checklist (CSC)

In agreement with the SCAT2 and the CNA chart, the University of Utah Sports Medicine Concussion Symptom Checklist covers 22 symptoms. The symptoms and the scoring are identical with the CNA symptom chart. Each concussed student athlete completed the checklist with a certified athletic trainer daily. The total symptom score was used in assessing the athlete’s progress and progression through the return-to-play protocol as defined in the University of Utah Sports Medicine Concussion Management Plan. When the symptom score was equal to zero, the player was no longer required to complete the symptom checklist. It was adopted from the CNA tool in an attempt to maintain continuity when comparing the symptom scores between the three different evaluation tools. This list and the CNA test could be completed over several different days, while the SCAT2 test was only administered at the time of a suspected SRC.

### BESS

The Balance Error Scoring System (BESS) is a balance test developed to provide clinicians with an inexpensive and practical tool for the assessment of postural stability [33]. The BESS is frequently used for the assessment of postural stability in concussed athletes [34]. The test is comprised of three parts: double-leg stance (hands on the hips and feet together), single-leg stance (standing on the non-dominant leg with hands on hips), and a tandem stance (non-dominant foot behind the dominant foot) in a heel-to-toe fashion. Each of the three parts is executed on a firm and on a foam surface (eyes closed, 20 s each). The errors are counted during each trial, defined as (i) lifting hands off iliac crest, (ii) opening eyes, (iii) stepping, stumbling or falling, (iv) remaining out of test position for more than 5 s, (v) moving the hip into more than 30° flexion or abduction, and (vi) lifting forefoot or heel [33].

### Statistics

Given the nature of this study and the small sample size, the authors chose to analyze the data in a largely descriptive manner to avoid a misrepresentation and misinterpretation of the results [4]. ANOVA was performed for biomarker significance determination followed by a Tukey HSD post hoc analysis. Statistical analysis and figure generation were accomplished using *R* [35] with the ggplot2 graphics package [36], respectively.

### Ethical considerations

This project and its protocols were approved by the University of Utah Institutional Review Board (protocol #00061977) and required informed consent for participation. All included athletes were consented at the time of the baseline sample blood draw event consistent with IRB requirements.

## Results

A comparison of the Symptom Charts currently used is provided in Table 1. In total, 12 SRCs in 11 athletes (20.6 ± 2.2 years) were reported during the football season between August and November in 2013. Nine SRCs were reported occurring during a practice session and three during a play. Seven SRCs were caused by a direct hit to the athlete’s head by another athlete’s helmet (six) or a head collision with an object (one). Two SCRs were reported after a hard hit to the body, and three SRC reports were made after an unknown course of events (two) and after an unrelated injury (one). Less than half of the SRCs were self-reported (five). The majority was discovered by either an athletic trainer (five) or by the position coach (two). On average, concussed athletes began the return-to-play protocol after 2 days (1.9 ± 2.3 days) with a range of 3–48 days. For reasons of safety, two athletes were medically disqualified to return to play. RTP decisions were made based on a previously reported strategy consistent with current recommendations and guidelines [12].

**Table 1:** Comparison of Symptom Charts used for concussion assessments

	Symptoms	SCAT2	CNA	CSC
General/cerebral	Nausea or vomiting	*	*	*
	Dizziness	*	*	*
	Sensitivity to light	*	*	*
	Sensitivity to noise	*	*	*
	Feeling slowed down	*	*	*
	Feeling like “in a fog”	*	*	*
	“Don’t feel right”	*		
	Fatigue or low energy	*	*	*
	Confusion	*		
	Drowsiness	*	*	*
Pain/missensation	Headache	*	*	*
	“Pressure in head”	*		
	Neck Pain	*		
	Numbness or tingling		*	*
Impaired function	Vision	*	*	*
	Balance	*	*	*
	Concentration	*	*	*
	Memory	*	*	*
	Sleep	*^a^	*^a,b^	*^a,b^
Emotions	More emotional	*	*	*
	Irritability	*	*	*
	Sadness	*	*	*
	Nervous or anxious	*	*	*
Other		*^c,d^		*^c^

The presented data compare the symptom charts of the Sport Concussion Assessment Tool 2 (SCAT2), the Computerized Neuropsychological Assessment (CNA), and the CSC (a symptom scale based on the University’s Concussion Program). All symptom charts rank and score the symptoms on a scale from 0 (none) to 6 (severe)

^a^Trouble falling asleep

^b^Sleeping more/less than usual

^c^Symptoms get worse with physical/mental activity? (Y/N)

^d^Is athlete acting different compared to the usual self? (no different/very different/unsure)

As of the current state evaluation and a critical aspect to understanding how best to implement biomarkers into the workflow, we evaluated the time difference between the date of injury and the post-injury assessment for the concussion assessment tools in use. Summary data for the time differences for each Symptom Chart are provided in Table 2. In addition, a summary of the time difference between injury and the initial venipuncture are included. The time deference data for injury assessments and venipuncture indicate a wide distribution range that is consistent with the variability in symptom presentation and time to reporting of a sustained injury. The mean and median time differences for the post-injury venipuncture indicate adequate compliance with expectations of a venipuncture occurring within 2 h of the reported injury; however, the wide range further highlights the difficulty with reliance on self-reporting.

**Table 2:** Time difference between the date of injury and post-injury assessments

	*Δ* DOI–DOA post	Mean ± STDEV	Median
BESS	0–7 d	1.8 ± 2.2 d	1.0 d
CNA	1–7 d	1.9 ± 1.8 d	1.0 d
SCAT2	15 min–158 hrs	28:26 ± 48:48 hrs	5:07 hrs
CSC	1–19 d	4 ± 5.5 d	2.0 d
Biomarker (S100B and NSE)	15 min–17:28 hrs	5 ± 7:08 hrs	2:00 hrs

This table displays the time difference (*Δ*) between the date of injury (DOI) and the date of the first concussion assessment following the injury (DOA post) using the most suitable unit of time in days (d), hours (hrs), and minutes (min). Mean and standard deviation are expressed in Mean ± STDV. The assessment includes the Balance Error Scoring System (BESS), Computerized Neuropsychological Assessment (CNA), the Sport Concussion Assessment Tool 2 (SCAT2), the CSC (a symptom scale based on the University’s Concussion Program) and the blood-based biomarkers S100 Beta (S100B) and neuron-specific enolase (NSE)

**Fig. 1:**
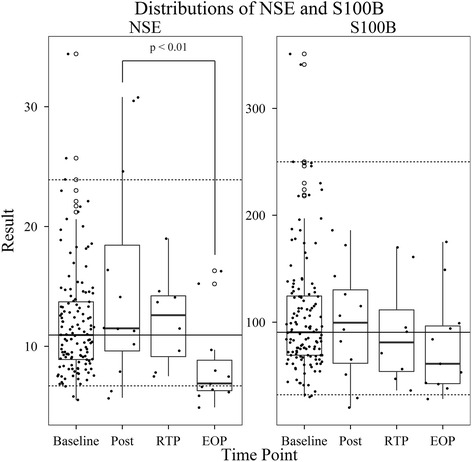
NSE and S100B results from the baseline, post-injury (post), return-to-play (RTP) and end-of-play (EOP) venipunctures. Upper and lower hinges correspond to the 25th and 75th percentiles, respectively. The upper whisker extends from the 75th percentile to the highest value within 1.5 times the interquartile range (IQR). The lower whisker extends from the 25th percentile to the lowest value within 1.5 times the IQR. Data beyond the whiskers are outliers and plotted as *open circles*. Individual data points (*solid black*) are overlaid. The *horizontal lower dashed line*, *solid black line*, and *upper dashed line* represent the 2.5, 50, and 97.5 non-parametric percentiles, respectively, of the baseline results. The established reference interval that includes the middle 95 % of the baseline data was determined to be 6.7 to 23.9 μg/L for NSE and 32 to 250 ng/L for S100B. A statistically significant difference was observed between the post and EOP venipunctures for NSE

One of the challenges in applying clinical testing to a unique population such as collegiate athletes is the lack of suitable reference intervals for categorizing patient results in an appropriate context. Further, previously established “cutoffs” used in determining clinical sensitivity and specificity are expected to vary widely among differing populations and clinical circumstances. Figure 1 provides a summary of the NSE and S100B results for the baseline, post-injury, return-to-play and end-of-play venipunctures. In order to provide a population appropriate reference interval, baseline values (*n* = 127) were used to establish a non-parametric reference interval using the 2.5and 97.5 % of the data for NSE and S100B. The established reference intervals were 6.7 to 23.9 μg/L for NSE and 32 to 250 ng/L for S100B. In comparison, reference intervals based upon healthy, non-athletes commonly used in clinical interpretations are 3.7 to 8.9 μg/L for NSE and 0 to 96 ng/L for S100B. A statistically significant difference was observed between the post-injury and end-of-play (EOP) results for NSE; however, a characteristic trend in the distribution of results for NSE and S100B was noted.

Although no clinical interpretation was applied to the NSE and S100B results during the season, we include the biomarker results in a summary table of outcome measures for athletes sustaining a diagnosed SRC in Table 3 and the individual results for each athlete in Table 4. Only the post-injury SCAT2 had 100 % completion for all diagnosed SRCs among the concussion assessment tools while all venipunctures were completed at the required time points. Of the 12 diagnosed concussions, 100 % (9/9) of the CNA and CSC assessments conducted at the RTP time point had lower symptom scores when compared to the post-concussive assessments consistent with approved protocols for RTP. For S100B, 50 % (6/6) had RTP or EOS results lower than the post-injury result with the other 50 % having higher results at the RTP or EOS time points when compared to the post-injury venipuncture. In contrast, 92 % of NSE results (11/12) were lower at RTP or EOP time points when compared to the corresponding post-injury venipuncture. Of note, for athletes #11 and #12 who were medically disqualified and unable to return to play, the S100B concentration remained consistent with the post-injury result while NSE decreased in concentration. Interpretation of the data using the established reference intervals resulted in 100 % of the baseline NSE and S100B values for the 12 injured athletes below the upper limit of the respective reference interval. For the post-injury samples, 100 % of the S100B results were within the established reference interval while 25 % (3/12) of the NSE results were above the upper limit of the reference interval. One hundred percent of the results for S100B and NSE were within the established reference intervals for the RTP and EOS samples.

**Table 3:** Combined outcome for concussion assessment tools and biomarker results for diagnosed SRCs

Tool	Unit	POM	*n*	Min	Max	Mean	STDEV	Median
BESS	Error	Baseline	11	4	25	12.2	5.0	11
BESS	Error	Post	11	5	37	16.7	9.3	13
CNA	SC	Baseline	12	0	15	3.3	5.6	0
CNA	SC	Post	11	0	89	25.0	28.3	18
CNA	SC	RTP	3	0	1	0.3	0.6	0
SCAT2	SC	Post	12	2	102	39.1	28.6	34
CSC	SC	Post	9	0	91	22.1	31.4	7
CSC	SC	RTP	7	0	2	0.3	0.8	0
S100B	ng/L	Baseline	12	56	174	100.9	40.1	89
S100B	ng/L	Post	12	20	186	99	52.9	100
S100B	ng/L	RTP	12	28	170	78.6	45.8	66
S100B	ng/L	EOS	12	28	149	80.4	45.8	73
NSE	μg/L	Baseline	12	5.8	21.7	13.5	5.1	14
NSE	μg/L	Post	12	5.7	30.8	15.1	8.8	12
NSE	μg/L	RTP	12	4.9	14.6	10.3	4.3	9
NSE	μg/L	EOS	12	4.9	16.3	9.2	4.3	7

The presented data provide an overview over the total outcome of the concussion assessment displayed by their unit (error during performance (Error), Symptom score (SC), nanogram per liter (ng/L), microgram per liter (μg/L), point of measurement (POM), sample size (*n*), minimum (Min), maximum (Max), mean (Mean), and standard deviation (STDEV). The assessment includes the score from the Balance Error Scoring System (BESS), the score of the symptom charts of the Computerized Neuropsychological Assessment (CNA), the Sport Concussion Assessment Tool 2 (SCAT2), and the CSC (a symptom scale based on the University’s Concussion Program), as well as the results of the blood-based biomarkers S100 Beta (S100B) and neuron-specific enolase (NSE)

**Table 4:** Individual outcome Symptom Charts

Tool	Unit	POM	1	2	3	4	5	6	7	8	9	10	11^a^	12^a^
Concussion symptom assessment														
BESS	Error	Baseline	25	4	14	*	13	11	10	11	11	10	11	14
BESS	Error	Post	30	11	20	*	37	14	13	13	11	18	5	12
CNA	SC	Baseline	4	0	0	6	15	0	14	0	0	0	0	0
CNA	SC	Post	30	0	40	1	18	58	10	3	0	*	26	89
CNA	SC	RTP	*	*	0	*	0	*	*	*	*	*	*	1
SCAT	SC	Post	46	9	102	23	27	66	2	30	12	50	38	64
CSC	SC	Post	21	0	*	1	6	58	*	4	7	11	*	91
CSC	SC	RTP	0	*	*	0	0	0	*	*	*	0	2	0
Biomarker														
S100B	ng/L	Baseline	69	72	75	85	136	138	174	111	111	56	140	44
S100B	ng/L	Post	172	51	29	126	115	186	143	65	106	93	82	20
S100B	ng/L	RTP	47	36	⊃	170	91	95	161	71	56	⊃	⊃	⊃
S100B	ng/L	EOS	42	38	43	53	94	149	175	99	99	61	84	28
NSE	ng/mL	Baseline	12.3	17.8	18.3	10.1	18.9	11.4	7.8	14.9	14.9	5.8	21.7	7.6
NSE	ng/mL	Post	30.8	11.5	7.9	11.5	10.2	24.6	14.1	16.4	30.5	6.3	11.3	5.7
NSE	ng/mL	RTP	14.6	7.8	⊃	9.6	7.5	14.1	11.5	13.7	19	⊃	⊃	⊃
NSE	ng/mL	EOS	6.4	6.2	6.9	6.6	7.5	9.7	15.2	16.3	16.3	8	5.9	4.9

The presented data show the individual outcome of the concussion assessment displayed by their unit (Error, Symptom score (SC), biomarker)), point of measurement (POM), sample size (*n*), minimum (Min), maximum (Max), mean (Mean), and standard deviation (STDV). The assessment includes the score from the Balance Error Scoring System (BESS), the score of the symptom charts of the Computerized Neuropsychological Assessment (CNA), the Sport Concussion Assessment Tool 2 (SCAT2), and the CSC (a symptom scale based on the University’s Concussion Program), as well as the results of the blood-based biomarkers S100 Beta (S100B) and neuron-specific enolase (NSE). Missing values are displayed with the symbol “*”. Some athletes were cleared to RTP at the EOS. Accordingly, the symbol “⊃” indicates that the samples were drawn at the same time

^a^Athletes with labeled IDs (ID^a^) were medically disqualified to RTP

## Conclusions

In this study, we document the use of an interdisciplinary workflow for venipuncture, on-site sample processing, and central laboratory testing for the duration of an entire collegiate football season. Not surprisingly, in following a single, entirely male collegiate team, the overall sample size was limited in the number of concussions. In addition, our results provide an evaluation of concussion assessment tools in use at the time of manuscript preparation which did not include imaging studies as part of the treatment protocol. Although the overall utility of the biomarkers chosen for inclusion in our study remains controversial, we have developed a successful strategy in addressing the practical aspect of biomarker integration into the routine concussion assessment workflow. Further, by relying on clinically available assays, we provide an interdisciplinary approach with biomarker results generated under the requirements of laboratory testing with well-defined criteria for accuracy, reproducibility, and assay performance not required in the research setting [12, 37].

Although the primary objective of the current study was not to address the utility of S100B or NSE in diagnosing SRC, the results of the baseline venipuncture and the reference intervals established from the dataset indicate that for both S100B and NSE, the currently used reference intervals are likely not applicable to athletes. This finding is a common occurrence in laboratory medicine that often prompts the necessary establishment of population specific reference intervals. In the context of physical activity, interpretation of established reference intervals can be further complicated [38, 39] and necessitates tight control over experimental design and sample collection for research as well as a well-defined protocol for eventual implementation.

The introduction of biomarkers into athletics for SRC diagnosis is a multi-faceted process and success of any proposed biomarker or biomarker panels will rest largely on the capability of successful implementation into the current concussion assessment workflows. Although much attention has been focused on biomarker discovery, the presented study was designed to investigate the practicality of biomarker implementation in a routine concussion assessment workflow including venipunctures at key time points before and after injury in addition to clinical assessments made for RTP for an entire collegiate football team.

### Expert recommendations

As the discovery of novel biomarkers and biomarker panels continues, successful implementation will rely on a thorough understanding of the constraints of the entire system and how best to enact required protocols. A continued need exists to identify and implement biomarkers with utility in all phases of the injury cycle with careful attention given to the practical limitations inherent to the nature of athletic competition.

## Additional file

Additional file 1: Table S1. Itemized venipuncture kits. (DOCX 14.2 kb)

## Abbreviations

BESS: Balance Error Scoring System
NSE: neuron-specific enolase
RPT: computerized neuropsychological assessment
RPT: return to play
SCAT2: Sport Concussion Assessment Tool 2
SRC: sports-related concussion
