# Supplementary material for: Utilization of the clinical laboratory for the implementation of concussion biomarkers in collegiate football and the necessity of personalized and predictive athlete specific reference intervals
Source: EPMA J. 2016 Jan 27;7(1):1. doi: 10.1186/s13167-016-0050-x (PMC4730649; doi:10.1186/s13167-016-0050-x)
Supplement: Additional file 1: Table S1. — Itemized venipuncture kits. (DOCX 14.2 kb) [file 13167_2016_50_MOESM1_ESM.docx]

**Supplementary Data**

Supplementary Table 1. Itemized venipuncture kits.

| **Item** | **Quantity** |
| --- | --- |
| Specimen Bag | 1 |
| Tourniquet | 1 |
| Gauze | 2 |
| Alcohol pads | 2 |
| Needles | 2 |
| Barrel | 1 |
| Red Top Tube | 1 |
| TTE Royal Blue | 1 |
| Band-Aid | 1 |
| Transport Tube | 2 |
| Disposable Pipette | 2 |
| Latex Gloves | 2 |
